# A Novel Cellulose-Supported Polymer Electrolyte with High Ionic Conductivity for Lithium Metal Batteries

**DOI:** 10.3390/molecules29235487

**Published:** 2024-11-21

**Authors:** Xuefei Cao, Mingyang Xin, Jiaxin Yin

**Affiliations:** 1 Criminal Investigation and Counter-Terrorism College, Criminal Investigation Police University of China, Shenyang 110854, China; caoxuefei@cipuc.edu.cn; 2 School of Chemistry, Northeast Normal University, Changchun 130024, China; koujn890@nenu.edu.cn

**Keywords:** lithium metal battery, PAAA polymer electrolyte, high-voltage cathode, high ionic conductivity

## Abstract

The traditional liquid electrolytes pose safety hazards primarily attributed to the flammability of organic solvent, whereas solid-state electrolytes can significantly enhance the safety of lithium-ion batteries. Polymer solid electrolytes are being considered as an effective solution due to their excellent flexibility and low cost, but they suffer low ionic conductivity or high interface impedance. Here, the ketone-containing allyl acetoacetate monomers were polymerized within the cellulose membrane via UV photopolymerization to prepare a cellulose-supported poly-allyl acetoacetate polymer electrolyte. The PAAA electrolyte shows the ion conductivity of 1.14 × 10^−4^ S cm^−1^ and the electrochemical stability window of 4.5 V. The Li symmetric battery can stably cycle for 1500 h at 0.1 mA cm^−2^. The LiFeO_4_‖Li cell achieves a discharge specific capacity of 160 mAh g^−1^ and demonstrates excellent cycling stability. Matching with Ni-rich cathodes also delivers decent performance. The designed polymer electrolyte with high ionic conductivity offers new ideas and directions for the development of future energy storage technology.

## 1. Introduction

Lithium-ion batteries (LIBs) have garnered substantial attention due to their vast and diverse applications across numerous fields, ranging from electric vehicles and portable electronic devices to energy storage systems and beyond [1,2,3,4,5]. Recently, lithium (Li) metal, which boast a high theoretical specific capacity of 3860 mAh g^−1^, has emerged as an alternative to the traditional graphite anode to increase the energy density of LIBs. This shift toward Li metals promises to significantly elevate the energy storage capabilities of LIBs [6,7,8,9]. Concurrently, solid-state electrolytes (SSEs) have been introduced to replace the flammable liquid electrolytes traditionally used in LIBs [10,11,12,13]. The assembled solid-state lithium metal battery exhibits high energy density and exceptional safety characteristics. Despite these promising attributes, SSEs are confronted with several challenges in practical applications [14]. One of the primary issues is the low ionic conductivity of SSEs at room temperature. For instance, common oxide-based SSEs exhibit room temperature ionic conductivity values ranging from approximately 10^−6^ to 10^−4^ S/cm, which is significantly lower than desired for optimal battery performance [15]. This limitation can adversely affect the rate capability and overall efficiency of the battery. The Nafion membrane exhibits good Li^+^ transport rates and selectivity [16], effectively mitigating the adverse effects of anion polarization during battery operation, which has been widely applied in flow batteries and lithium-sulfur batteries, and attempted in LIBs [17]. Nevertheless, Nafion-type membranes confront challenges, including production difficulties, high costs, strict synthesis conditions concerning temperature and moisture, and substantial obstacles in their integration into LIBs. Furthermore, the rigid nature of SSEs often leads to poor interface contact with the solid electrodes [10,18,19], which tends to result in higher interface resistance and even becomes a barrier to the widespread adoption of SSEs in LIBs. To overcome these challenges, researchers are actively exploring various strategies, such as optimizing the composition and structure of SSEs, and enhancing the electrode-electrolyte interface through innovative materials or designs [20,21,22]. These efforts are crucial for the continued development and improvement of solid-state lithium metal batteries.

Through extensive research and practice, it has become well-established that polymer electrolytes can effectively mitigate the issue of solid-solid interface contact, thanks to their high flexibility, low interface impedance with electrode materials [23,24,25]. Despite these advantages, the low ion conductivity of polymer electrolytes remains a significant challenge, hindering their practical applications. For instance, the room temperature ion conductivity of the well-known Polyethylene Oxide (PEO)-based electrolyte is merely 10^−7^ S cm^−1^ [26]. To tackle this issue, various strategies have been proposed. One approach involves structural modification of polymers to incorporate more groups that contribute to ion transport [27,28]. Xie’s group, for example, reduced the crystallization of PEO by copolymerizing it with other monomers, resulting in a disordered and ordered electrolyte with an ion conductivity of 2.7 × 10^−4^ S cm^−1^ [29]. Another method involves incorporating high-ionic-conductivity inorganic solid electrolytes as active ceramic fillers. These fillers, along with the polymer and the polymer-ceramic interface, collectively facilitate ionic transport, thereby enhancing the ionic conductivity of the composite solid-state electrolyte [30]. However, this approach can lead to a poorer ion transport interface environment, making it difficult for lithium ions to undergo two-phase exchange and resulting in slower two-phase diffusion [31]. In recent years, carbonyl-based polymers have been discovered and applied in lithium-ion batteries. The carbonyl (-C=O) group in these polymers facilitates the dissociation of lithium salts, enhancing their performance [32]. In addition to containing ester groups, allyl acetoacetate (AAA) also exhibits ether groups that have weaker complexation with Li^+^, leading to a faster intercalation/de-intercalation process. Zhang et al. attempted to blend AAA with PEO to enhance the stability of the electrolyte film with Li metal, but the resulting film was relatively thick [33]. To further improve the ionic conductivity of AAA, Li and his team added SiO_2_ to disrupt its crystallization [34]. As a result, AAA has emerged as a more promising electrolyte in recent years, offering new possibilities for the development of high-performance lithium-ion batteries.

Here, we successfully synthesized the PAAA electrolytes through the in situ polymerization of AAA monomers. Utilizing a cellulose film as the support structure not only significantly reduces the thickness of the electrolyte film but also effectively prevents the formation of lithium dendrites to a considerable degree. Consequently, the resultant electrolyte exhibits an impressive ion conductivity of 1.41 × 10^−4^ S cm^−1^, which fully satisfies the operational requirements of lithium-ion batteries. Furthermore, the lithium-ion migration number is 0.81, and the electrochemical oxidation window reaches 4.5 V, suggesting the feasibility of pairing this electrolyte with Ni-rich electrodes. When matched with a LiFeO_4_ (LFP) electrode, the discharge specific capacity achieves 160 mAh g^−1^, with a capacity retention rate of 95% after 200 cycles. Notably, the PAAA demonstrates exceptional rate performance, maintaining capacities of 158 mAh g^−1^, 152 mAh g^−1^, 146 mAh g^−1^, and 123 mAh g^−1^ at 0.1 C, 0.2 C, 0.5 C, and 1 C, respectively. Additionally, the Li-Li cells utilizing PAAA can stably cycle for 1500 h at 0.1 mA cm^−2^ and 0.1 mAh cm^−2^. Our synthesized PAAA electrolyte thus offers a promising new pathway for the construction of all-solid-state lithium metal batteries, paving the way for advancements in energy storage technology.

## 2. Results

### 2.1. Synthesis and Characterizations of PAAA

**Figure 1:**
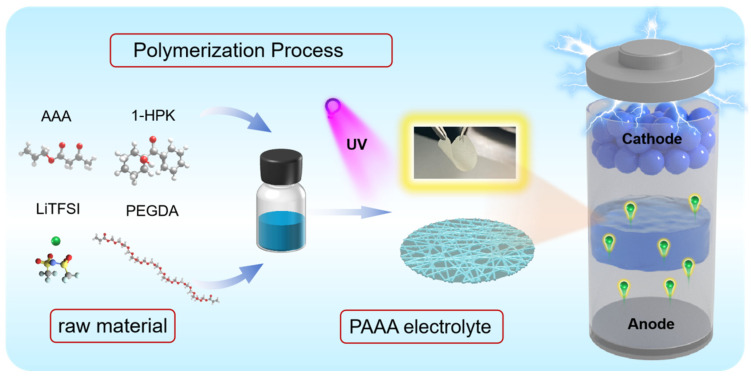
The schematic illustration of synthetic procedure of the PAAA composite electrolyte.

We have developed a novel design for a PAAA (poly(allyl acetoacetate)) composite electrolyte through a straightforward UV photo-polymerization process, as illustrated in Figure 1. In this process, AAA (allyl acetoacetate) serves as the monomer, while PEGDA (poly(ethylene glycol) diacrylate) acts as the cross-linker. These components undergo polymerization within a cellulose membrane to prepare a PAAA composite electrolyte that boasts an interpenetrating network structure. Crucially, the polymers that undergo polymerization in the absence of the cellulose membrane exhibit brittleness (as shown in Figure S1), making it challenging to form a self-supporting membrane. However, by incorporating the cellulose membrane we are able to achieve a composite electrolyte membrane with a thickness of only 35 μm. The membrane not only maintains the structural integrity of the electrolyte but also benefits from the inherent properties of cellulose. Previous studies have indicated that cellulose can, to some extent, impede the penetration of lithium dendrites [35]. Lithium dendrites would cause internal shorts and ultimately lead to battery failure. By incorporating cellulose into our PAAA composite electrolyte, we aim to further enhance its safety and reliability by mitigating the risk of dendrite formation. Overall, our novel design for the PAAA composite electrolyte not only addresses the brittleness issue of the polymerized material but also leverages the benefits of cellulose to improve the overall performance and safety of the electrolyte.

**Figure 2:**
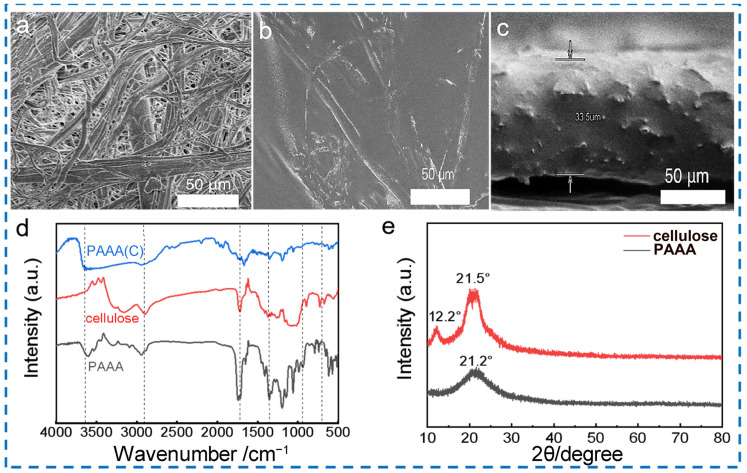
SEM images for (**a**) cellulose and (**b**) PAAA composite electrolyte, (**c**) cross-sectional SEM images for PAAA composite electrolyte, (**d**) FTIR spectra and (**e**) XRD pattern.

The SEM (scanning electron microscope) images presented in Figure 2a and b offer a clear comparison between the cellulose and the PAAA composite electrolyte. The cellulose membrane exhibits a loose and porous surface structure. In contrast, the PAAA composite electrolyte, depicted in Figure 2b, fills the pores of the cellulose uniformly, resulting in a relatively flat and smooth surface with no obvious pores visible. This uniform infiltration of the polymer electrolyte into the cellulose matrix indicates a successful integration of the two components. Furthermore, nitrogen sorption tests were conducted to determine the porosity of cellulose membrane and PAAA. The adsorption and desorption isotherm curves were shown Figure S2. BET analyses revealed a surface area of 0.97 m^2^ g^−1^ for cellulose and 0.22 m^2^ g^−1^ for PAAA, indicating that the PAAA has almost been filled into the cellulose pores, which is consistent with the SEM characterization. As illustrated in Figure 2c, a uniform and dense layer of the PAAA composite electrolyte film is observed, with a thickness of approximately 33.5 μm. This confirms the simplicity and effectiveness of the synthesis method, as well as the practicality of the electrolyte membrane’s thickness for potential applications.

To further analyze the microstructure of the PAAA composite electrolyte, Fourier Transform Infrared Spectrometer (FTIR) analysis was conducted to observe changes in the functional groups of supported-PAAA, PAAA(C), and cellulose. As depicted in Figure 2d, no significant changes were observed in the FTIR spectra of PAAA and PAAA(C), such as the presence of the C-F bond at 1220 cm⁻^1^ and the S-O-S bond at 1350 cm⁻^1^. This suggests that the chemical structure of the PAAA composite electrolyte remains stable and consistent [33]. Additionally, X-Ray Diffraction (XRD) results further indicated that the polarization of the PAAA precursor within the cellulose membrane skeleton did not lead to significant changes in the crystal structure of the composite [36]. This stability in the crystal structure is crucial for maintaining the functional properties of the electrolyte. To assess the thermal behavior of the PAAA composite electrolyte, Thermal Gravimetric Analyzer (TGA) analysis was performed. The TGA curve (Figure S3) shows that the weight loss of the film begins to occur before 300 °C, which corresponds to the degradation of AAA. Despite this, the thermal decomposition temperature of the PAAA composite electrolyte is suitable for application in lithium metal batteries, ensuring that the functional advantages of the as-prepared electrolyte are maintained. The above results and analysis provide a comprehensive understanding of the microstructure, chemical stability, and thermal behavior of the PAAA composite electrolyte. These findings further validate the effectiveness and practicality of the synthesis method, as well as the potential applications of the composite electrolyte in lithium metal batteries.

### 2.2. Electrochemical Properties of PAAA

**Figure 3:**
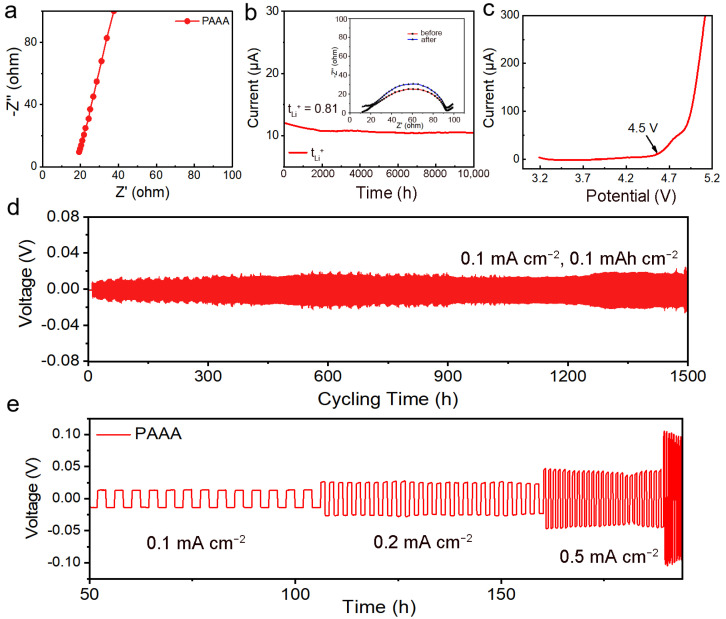
(**a**) The electrochemical impedance spectroscopy (EIS) curve of PAAA at room temperature, (**b**) CA curve and AC impedance spectroscopy before and after polarization of PAAA, (**c**) the linear sweep voltammetry (LSV) curve of PAAA; the time–voltage curve of Li-Li cell (**d**) at 0.1 mA cm^−2^ and 0.1 mAh cm^−2^, and (**e**) at various current densities.

The electrochemical performance of the PAAA composite electrolyte was thoroughly evaluated to assess its suitability for application in LIBs. Initially, electrochemical impedance spectroscopy (EIS) tests were conducted to determine the ionic conductivity of the PAAA composite electrolyte. As depicted in Figure 3a, the impedance of the PAAA composite electrolyte was measured to be 25 Ω. Based on this impedance value, the room temperature ionic conductivity of the electrolyte was calculated to be 1.41 × 10^−4^ (Equation (S1)). This high ionic conductivity indicates that the PAAA composite electrolyte has the potential for application in LIBs, where high conductivity is crucial for efficient battery performance. In addition to ionic conductivity, the transfer number of lithium ions (t_Li_^+^) is a critical parameter for the application of solid-state electrolytes in Li metal batteries. To measure the t_Li_^+^ of the PAAA composite electrolyte, the constant voltage polarization method was employed. The initial polarization current (I_0_) and the steady-state polarization current (I_SS_) were recorded as 12 μA and 10 μA, respectively, as shown in Figure 3b. Using the Bruce–Vincent–Evans equation, the t_Li_^+^ was calculated to be 0.81. This high t_Li_^+^ value suggests that the PAAA composite electrolyte exhibits superior performance in terms of lithium ion mobility and lithium ion conductivity, which are essential for efficient Li metal battery operation [37]. Furthermore, the electrochemical stability window (ESW) of the electrolyte was evaluated using linear sweep voltammetry (LSV). A high ESW was critical for ensuring that the electrolyte can match the high-voltage electrode, which is required for achieving high energy density in LIBs. As illustrated in Figure 3c, the PAAA composite electrolyte exhibited no anodic current until it reached 4.5 V. This result indicates that the PAAA composite electrolyte has a high resistance to oxidation and potential compatibility with high-voltage cathodes, which is essential for the development of high-performance LIBs. In summary, the PAAA composite electrolyte demonstrated excellent electrochemical performance, including ionic conductivity, lithium ion mobility, and electrochemical stability window. These results suggest that the PAAA composite electrolyte has significant potential for application in LIBs, where it can contribute to enhanced battery performance and energy density.

Li-Li symmetric cells were assembled to rigorously evaluate the stability of the electrolytes on lithium metal anodes. As shown in Figure 3d, these symmetrical batteries exhibited remarkable stability during cycling, maintaining a constant overpotential of 0.02 V for an impressive 1500 cycles at a current density of 0.1 mA cm^−2^. This stability was further confirmed by testing the cells at higher current densities, as illustrated in Figure 3e, where the overpotential remained stable even under more demanding conditions. To gain further insight into the stability of the electrolyte film towards lithium metal, SEM images were taken (Figure S4) after cycling. These observations revealed that the surface of the lithium metal remained smooth and pristine, with no signs of dead lithium or dendrite formation. The absence of dendrites in our tests confirms the effectiveness of the designed electrolyte film in stabilizing the lithium metal anode and preventing dendrite growth [38]. In conclusion, the Li-Li symmetric cell tests demonstrate the exceptional stability of the electrolyte film towards lithium metal, with stable cycling performance and smooth lithium metal surfaces after cycling. These results further validate the potential of the PAAA composite electrolyte for use in lithium metal batteries, where stability and performance are paramount.

### 2.3. Battery Cycling Performance

**Figure 4:**
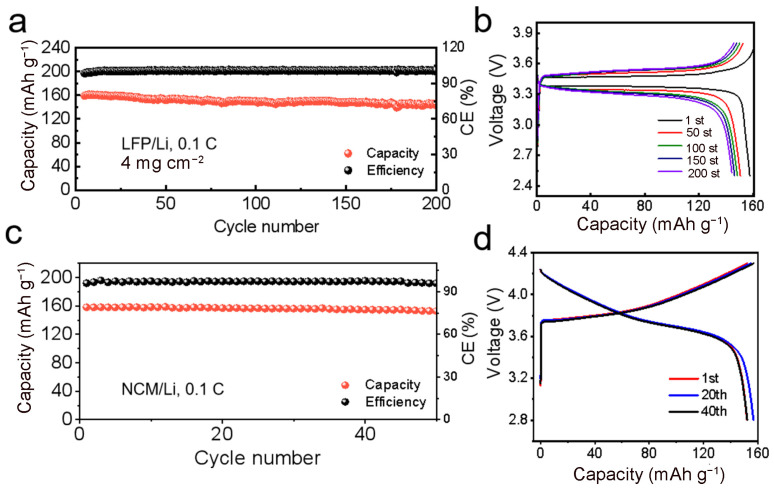
(**a**) The cycle performance of Li‖LFP full cells at 0.1 C, (**b**) the charge/discharge curve at various cycles, (**c**) the cycle performance of Li‖NCM full cells at 0.1 C and (**d**) the charge/discharge curve at 1st, 20th 40th.

The electrochemical performance of the PAAA polymer electrolyte was rigorously evaluated through galvanostatic charge/discharge measurements of Li‖LFP full cells. As illustrated in Figure 4a, the Li‖LFP cell demonstrated an impressive initial discharge capacity of 162 mAh g^−1^, and it retained a high capacity of 90% after 200 cycles, accompanied by an exceptional average coulombic efficiency of 99.9%, superior to previously reported ketone-based electrolytes [33,39]. The charge/discharge curve in Figure 4b showcased a typical and stable charge and discharge platform throughout the cycling process, with a remarkably low polarization voltage of 0.21 V. The cycling stability can be attributed to the high ion conductivity of the PAAA electrolyte and its excellent compatibility with Li metal. Furthermore, the Li‖LFP cell exhibited outstanding rate performance, achieving capacities of 158 mAh g^−1^, 152 mAh g^−1^, 146 mAh g^−1^, and 123 mAh g^−1^ at rates of 0.1 C, 0.2 C, 0.5 C, and 1 C, respectively. Notably, even after the rate was adjusted back to 0.1 C, the cell regained its capacity of 157 mAh g^−1^ and continued to cycle stably, as shown in Figure S5. This excellent rate performance can be attributed to the high lithium-ion conductivity (t_Li_^+^) of the PAAA electrolyte, which allows for efficient ion transport and rapid charge-discharge processes.

To further elevate the energy density of the battery, we embarked on an initiative to assemble it by meticulously matching the electrolyte with a Ni-rich cathode material. This pairing was aimed at harnessing the high capacity and energy density potential of Ni-rich cathodes while ensuring compatibility with our PAAA composite electrolyte. The initial discharge capacities of the PAAA Li‖NCM cells, tested at a rate of 0.1C and room temperature within a voltage window of 2.8–4.3 V, yielded results of 159.7 mAh g^−1^, as depicted in Figure 4c. Beyond the initial discharge capacity, the performance of the Li‖NCM cell was further evaluated through cycling stability tests. After enduring 50 cycles, the cell exhibited minimal polarization potential, which is indicative of its ability to maintain efficient charge and discharge processes. This minimal polarization can be attributed to the acceptable equivalent series resistance (ESW) of the cell, reflecting the excellent interface stability between the PAAA composite electrolyte and the Ni-rich cathode material. Above results not only validate the feasibility of the PAAA composite electrolyte design for application in lithium batteries but also underscore its potential to enhance the energy density of such batteries when paired with high-capacity Ni-rich cathode materials. The minimal polarization and stable cycling performance observed in the Li‖NCM cells suggest that the PAAA composite electrolyte is a promising candidate for use in advanced lithium-ion batteries aiming for higher energy densities and improved performance.

### 2.4. Interface Analysis

**Figure 5:**
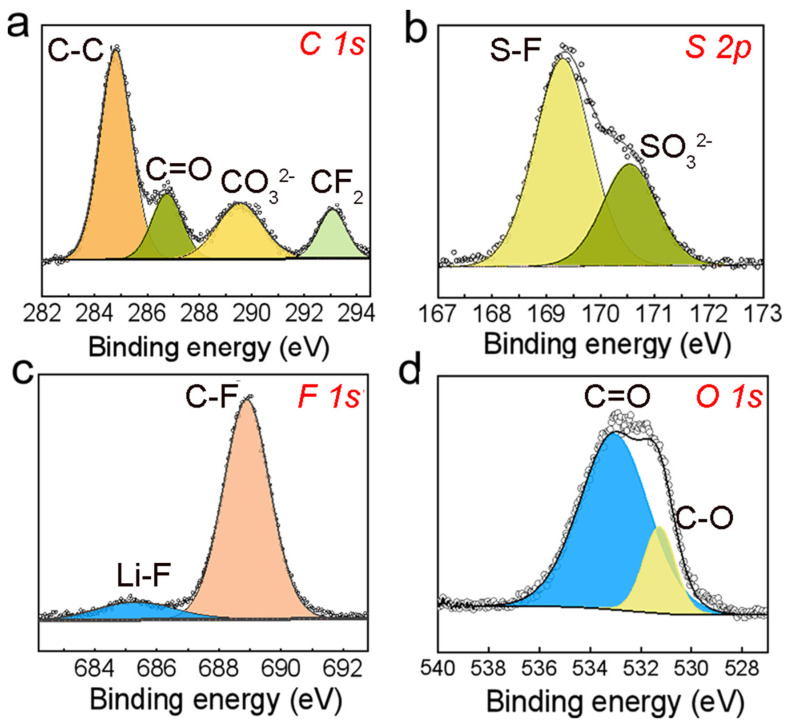
The XPS fine spectra of (**a**) S 2p, (**b**) O 1s, (**c**) F 1s, and (**d**) C 1s.

To further assess the stability of PAAA and Li metal, comprehensive analytical techniques were employed, including X-ray photoelectron spectroscopy (XPS), which was used on Li metal extracted from the cycled NCM/PAAA-C/Li cell. This analysis was complemented by a detailed examination of the solid-electrolyte interphase (SEI) components. Figure 5 presents the XPS fine spectra of key elements within the SEI, namely S 2p, O 1s, F 1s, and C 1s. The C 1s spectrum reveals four distinct peaks, each corresponding to specific chemical bonds within the SEI. The peak at 284.7 eV is attributed to C-C bonds, indicating the presence of carbonaceous materials. The peak at 286.3 eV corresponds to C=O bonds, which represent the organic components of the SEI, likely derived from the decomposition of PAAA. The peak at 288.5 eV is associated with Li_2_CO_3_, a compound that enhances the mechanical robustness of the SEI [40]. Lastly, the peak at 292.6 eV is linked to -CF_2_ bonds, reflecting the incorporation of fluorine-containing species [41]. The S 2p spectrum displays peaks at 168.7 eV and 171 eV, which correspond to S-F and SO_3_^2−^ bonds, respectively. These bonds are the result of the decomposition of LiTFSI, a salt commonly used in lithium-ion batteries [42]. The absence of additional peaks suggests that no undesirable side reactions occurred during the cycling process, indicating the chemical stability of the SEI. Furthermore, the F 1s spectrum exhibits characteristic peaks at 688.5 eV and 684.8 eV, which are attributed to LiTFSI and LiF, respectively. Notably, LiF serves as a pivotal buffer layer within the SEI, effectively inhibiting the growth of lithium dendrites [43]. This is due to LiF’s electronic insulating properties, which protect the lithium metal anode by blocking electron transport and mitigating dendrite formation. Collectively, these results demonstrate that an organic-inorganic composite SEI is formed on the surface of lithium after cycling. This composite SEI, which combines the mechanical stability of inorganic components with the flexibility of organic components, further enhances the cycling stability of the battery. By providing a robust protective layer that minimizes side reactions and inhibits dendrite growth, the SEI formed in this study contributes to the overall stability and performance of the NCM/PAAA-C/Li cell.

## 3. Materials and Methods

### 3.1. Preparation of PAAA Membranes

The monomer allyl acetoacetate (AAA, 97%, procured from Aladdin, Bay City, MI, USA), lithium bis(trifluoromethylsulfonyl)imide (LiTFSI, 99%, Alpha Aesar, Haverhill, MA, USA), cross-linking agent poly(ethylene glycol) bis(acrylate) (PEGDA, Mn = 600 g mol^−1^ and 1000 g mol^−1^, Aladdin), photoinitiator 1-hydroxy-cyclohexyl-phenyl ketone (98%, Aladdin) and cellulose membrane were used to prepare poly-AAA cellulose membrane (PAAA). A 0.73 g cross-linker PEGDA, 3.2 g LiTFSI, and 5 mL monomer AAA were mixed and stirred for 5 h at room temperature. Then, photoinitiator 1-hydroxy-cyclohexyl-phenyl ketone was added to the above uniform solution and stirred for 10 min. The mixture was coated with a squeegee of 60 µm on the front and back of cellulose membranes. The coated films were exposed to UV light at 365 nm for 5 min to initiate the polymerization reaction, and then baked in a vacuum oven at 60 °C with 133 Pa for 24 h to obtain the PAAA. The prepared solid polymer electrolyte film needs to be saved in an argon filled glove box for battery packing and testing.

### 3.2. Structure and Morphology Characterization

The solid polymer electrolyte film underwent a series of rigorous analytical tests to fully characterize its properties. Scanning Electron Microscopy (SEM) was employed to investigate the structure and morphology of the material. This test allowed for a detailed examination of the film’s surface texture, porosity, and any potential defects or irregularities. Fourier Transform Infrared Spectroscopy (FTIR) was used to analyze the chemical bonding composition of the solid electrolyte film. This technique provided insights into the functional groups and chemical bonds present within the material, helping to understand its composition and potential reactivity. Thermogravimetric analysis (TGA) was conducted to evaluate the thermal stability of the solid electrolyte film. This test analyzed the decomposition behavior of the material at high temperatures, revealing its thermal stability limits and potential degradation mechanisms. Furthermore, X-ray Diffraction (XRD) was utilized to observe the crystal structure of the solid electrolyte film. This technique provided information on the crystallinity, phase purity, and lattice parameters of the material, which are crucial for understanding its electrochemical performance and stability. The Brunauer–Emmett–Teller (BET) surface area and total pore volumes were calculated from the N_2_ sorption isotherms at 77 K, and the pore size distribution was calculated based on the N_2_ sorption isotherm using density functional theory.

### 3.3. Electrochemical Performance Testing

During the electrochemical testing, the glove box was maintained under an argon atmosphere with water and oxygen concentrations below 0.01 ppm. Within this controlled environment, 2025-type button cells were assembled and subjected to a series of rigorous tests. Constant-current charge–discharge evaluations were conducted using a Neware battery testing system, with LiFePO_4_/PAAA/Li cells tested within a voltage range of 2.5–3.8 V at various rates of 0.1, 0.2, 0.5, and 1.0 C. NCM/PAAA/Li cells were tested within a voltage range of 2.5–3.8 V at 0.1 C and room temperature. Subsequently, these cells were conducted on further constant-current charge–discharge testing utilizing a P4000 electrochemical testing system. EIS tests were performed on a P4000 electrochemical workstation at a frequency of 100 kHz–0.1 Hz. Linear scanning voltammetry (LSV) tests were performed over a voltage range of 0-6 V.

## 4. Conclusions

In summary, we have successfully prepared cellulose-supported PAAA polymer electrolyte membrane that exhibits high ion conductivity and wide electrochemical stability windows. The PAAA also possesses excellent thermal stability and tensile properties due to supported structure of cellulose. Consequently, LiFePO_4_‖Li cells utilizing PAAA electrolyte demonstrate a discharge specific capacity of up to 160 mAh g^−1^, with a capacity retention rate of 95% after 200 cycles. Furthermore, the NCM‖Li cells with PAAA also achieved accepted performance and great potential. Additionally, the Li-Li symmetrical battery can cycle stably for 1500 h, highlighting its good compatibility and stability with lithium metal. This study offers fresh methods and insight on the advancement of polymer electrolytes. More importantly, it can ensure stable performance of high-energy solid-state lithium batteries for applications in electric vehicles, public security equipment, and other scenarios.

## Data Availability

Data are contained within the article and Supplementary Materials.

## Supplementary Materials

The following supporting information can be downloaded at https://www.mdpi.com/article/10.3390/molecules29235487/s1, Figure S1: polymerization of PAAA without a cellulose membrane; Figure S2: nitrogen adsorption isotherm and desorption isotherm; Figure S3: thermogravimetry curves of PAAA; Figure S4: the SEM images from Li-Li cells after cycling; Figure S5: the capacity-efficiency plots of Li‖PAAA‖LFP at various rates.

## References

[1] Sun W. (2018). Diagnose lithium battery. Nat. Nanotechnol..

[2] Eftekhari A. (2018). High-Energy Aqueous Lithium Batteries. Adv. Energy Mater..

[3] Ponnada S., Kiai M., Krishnapriya R., Singhal R., Sharma R. (2022). Lithium-Free Batteries: Needs and Challenges. Energy Fuels.

[4] Bandini G., Caposciutti G., Marracci M., Buffi A., Tellini B. (2022). Characterization of lithium-batteries for high power applications. J. Energy Storage.

[5] Wei Q., Wu Y., Li S., Chen R., Ding J., Zhang C. (2023). Spent lithium ion battery (LIB) recycle from electric vehicles: A mini-review. Sci. Total Environ..

[6] Wang Q., Liu B., Shen Y., Wu J., Zhao Z., Zhong C., Hu W. (2021). Confronting the Challenges in Lithium Anodes for Lithium Metal Batteries. Adv. Sci..

[7] Ahmed R.A., Carballo K.V., Koirala K.P., Zhao Q., Gao P., Kim J., Anderson C.S., Meng X., Wang C., Zhang J. (2024). Lithicone-Protected Lithium Metal Anodes for Lithium Metal Batteries with Nickel-Rich Cathode Materials. Small Struct..

[8] Zhang Y., Zuo T.-T., Popovic J., Lim K., Yin Y.-X., Maier J., Guo Y.-G. (2020). Towards better Li metal anodes: Challenges and strategies. Mater. Today.

[9] Zhang B., Ma J.-P., Zhao Y., Li T., Yang J.-L., Zhang Z.-L., Wei S.-Z., Zhou G.-M. (2024). Design and application of copper/lithium composite anodes for advanced lithium metal batteries. Rare Met..

[10] Chai S., He Q., Zhou J., Chang Z., Pan A., Zhou H. (2024). Solid-State Electrolytes and Electrode/Electrolyte Interfaces in Rechargeable Batteries. ChemSusChem.

[11] Wu J., Rao Z., Wang H., Huang Y. (2022). Order-structured solid-state electrolytes. SusMat.

[12] He L., Oh J.A.S., Chua J.J.J., Zhou H. (2020). Solid-state electrolytes: Advances and perspectives. Funct. Mater. Lett..

[13] Gao X., Xing Z., Wang M., Nie C., Shang Z., Bai Z., Dou S.X., Wang N. (2023). Comprehensive insights into solid-state electrolytes and electrode-electrolyte interfaces in all-solid-state sodium-ion batteries. Energy Storage Mater..

[14] Liang X., Wang L., Wu X., Feng X., Wu Q., Sun Y., Xiang H., Wang J. (2022). Solid-state electrolytes for solid-state lithium-sulfur batteries: Comparisons, advances and prospects. J. Energy Chem..

[15] Kim K.J., Balaish M., Wadaguchi M., Kong L., Rupp J.L.M. (2021). Solid State Batteries: Solid-State Li–Metal Batteries: Challenges and Horizons of Oxide and Sulfide Solid Electrolytes and Their Interfaces (Adv. Energy Mater. 1/2021). Adv. Energy Mater..

[16] Sun C., Negro E., Nale A., Pagot G., Vezzù K., Zawodzinski T.A., Meda L., Gambaro C., Di Noto V. (2021). An efficient barrier toward vanadium crossover in redox flow batteries: The bilayer [Nafion/(WO_3_)x] hybrid inorganic-organic membrane. Electrochim. Acta.

[17] Bian T., Wang X., Zhang Q., Zhu X., Jiao J., Hou Z., Han Q., Guo Z., Wen L., Jiang L. (2024). Uniform Nanoscale Ion-Selective Membrane Prepared by Precision Control of Solution Spreading and Evaporation. Nano Lett..

[18] Ma M., Zhang M., Jiang B., Du Y., Hu B., Sun C. (2023). A review of all-solid-state electrolytes for lithium batteries: High-voltage cathode materials, solid-state electrolytes and electrode–electrolyte interfaces. Mater. Chem. Front..

[19] Chen X., Ye Y., Feng S., Lv B., Wang J., Tang J., Dou W. (2024). Improving Buried Interface Contact by Bidentate Anchoring for Inverted Perovskite Solar Cells. Small.

[20] Lu Y., Li L., Zhang Q., Niu Z., Chen J. (2018). Electrolyte and Interface Engineering for Solid-State Sodium Batteries. Joule.

[21] Palacin M.R. (2021). Battery Materials Design Essentials. Acc. Mater. Res..

[22] Yu X., Manthiram A. (2018). Electrode–electrolyte interfaces in lithium-based batteries. Energy Environ. Sci..

[23] Long L., Wang S., Xiao M., Meng Y. (2016). Polymer electrolytes for lithium polymer batteries. J. Mater. Chem. A.

[24] Choo Y., Halat D.M., Villaluenga I., Timachova K., Balsara N.P. (2020). Diffusion and migration in polymer electrolytes. Prog. Polym. Sci..

[25] Isikli S., Ryan K.M. (2020). Recent advances in solid-state polymer electrolytes and innovative ionic liquids based polymer electrolyte systems. Curr. Opin. Electrochem..

[26] Han L., Wang J., Mu X., Wu T., Liao C., Wu N., Xing W., Song L., Kan Y., Hu Y. (2021). Controllable magnetic field aligned sepiolite nanowires for high ionic conductivity and high safety PEO solid polymer electrolytes. J. Colloid Interface Sci..

[27] Fernando I.P.S., Kim D., Nah J.-W., Jeon Y.-J. (2019). Advances in functionalizing fucoidans and alginates (bio)polymers by structural modifications: A review. Chem. Eng. J..

[28] Bhat S.I., Ahmadi Y., Ahmad S. (2018). Recent Advances in Structural Modifications of Hyperbranched Polymers and Their Applications. Ind. Eng. Chem. Res..

[29] Zhang Y., Lu W., Cong L., Liu J., Sun L., Mauger A., Julien C.M., Xie H., Liu J. (2019). Cross-linking network based on Poly(ethylene oxide): Solid polymer electrolyte for room temperature lithium battery. J. Power Sources.

[30] Ranque P., Zagórski J., Devaraj S., Aguesse F., López del Amo J.M. (2021). Characterization of the interfacial Li-ion exchange process in a ceramic–polymer composite by solid state NMR. J. Mater. Chem. A.

[31] Liu M., Wang C., Zhao C., van der Maas E., Lin K., Arszelewska V.A., Li B., Ganapathy S., Wagemaker M. (2021). Quantification of the Li-ion diffusion over an interface coating in all-solid-state batteries via NMR measurements. Nat. Commun..

[32] Wang H., Yao C.-J., Nie H.-J., Wang K.-Z., Zhong Y.-W., Chen P., Mei S., Zhang Q. (2020). Recent progress in carbonyl-based organic polymers as promising electrode materials for lithium-ion batteries (LIBs). J. Mater. Chem. A.

[33] Chen P., Zeng Q., Li Q., Zhao R., Li Z., Wen X., Wen W., Liu Y., Chen A., Li Z. (2022). A ketone-containing all-solid-state polymer electrolyte with rapid Li-ion conduction for lithium metal batteries. Chem. Eng. J..

[34] Zhao Y., Li L., Zhou D., Ma Y., Zhang Y., Yang H., Fan S., Tong H., Li S., Qu W. (2024). Opening and Constructing Stable Lithium-ion Channels within Polymer Electrolytes. Angew. Chem. Int. Ed..

[35] Xin M., Lian X., Gao X., Xu P., Li W., Dong F., Zhang A., Xie H., Liu Y. (2023). Enabling high-capacity Li metal battery with PVDF sandwiched type polymer electrolyte. J. Colloid Interface Sci..

[36] Zhao C., Liu J., Li B., Ren D., Chen X., Yu J., Zhang Q. (2020). Multiscale Construction of Bifunctional Electrocatalysts for Long-Lifespan Rechargeable Zinc–Air Batteries. Adv. Funct. Mater..

[37] Wang Y., Sun Q., Zou J., Zheng Y., Li J., Zheng M., Liu Y., Liang Y. (2023). Simultaneous High Ionic Conductivity and Lithium-Ion Transference Number in Single-Ion Conductor Network Polymer Enabling Fast-Charging Solid-State Lithium Battery. Small.

[38] Zhang S., Cheng B., Fang Y., Dang D., Shen X., Li Z., Wu M., Hong Y., Liu Q. (2022). Inhibition of lithium dendrites and dead lithium by an ionic liquid additive toward safe and stable lithium metal anodes. Chin. Chem. Lett..

[39] Eriksson T., Mace A., Manabe Y., Yoshizawa-Fujita M., Inokuma Y., Brandell D., Mindemark J. (2020). Polyketones as Host Materials for Solid Polymer Electrolytes. J. Electrochem. Soc..

[40] Liu X., Mariani A., Diemant T., Di Pietro M.E., Dong X., Mele A., Passerini S. (2024). Reinforcing the Electrode/Electrolyte Interphases of Lithium Metal Batteries Employing Locally Concentrated Ionic Liquid Electrolytes. Adv. Mater..

[41] Zhang M., Liu R., Wang Z., Xing X., Liu Y., Deng B., Yang T. (2020). Electrolyte additive maintains high performance for dendrite-free lithium metal anode. Chin. Chem. Lett..

[42] Park K., Yu S., Lee C., Lee H. (2015). Comparative study on lithium borates as corrosion inhibitors of aluminum current collector in lithium bis(fluorosulfonyl)imide electrolytes. J. Power Sources.

[43] Yu T., Zhao T., Zhang N., Xue T., Chen Y., Ye Y., Wu F., Chen R. (2023). Spatially Confined LiF Nanoparticles in an Aligned Polymer Matrix as the Artificial SEI Layer for Lithium Metal Anodes. Nano Lett..

