# Supplementary material for: A Novel Cellulose-Supported Polymer Electrolyte with High Ionic Conductivity for Lithium Metal Batteries
Source: Molecules. 2024 Nov 21;29(23):5487. doi: 10.3390/molecules29235487 (PMC11643007; doi:10.3390/molecules29235487)
Supplement: Supplementary file 1 [file molecules-29-05487-s001.zip › molecules-3294927-supplementary.pdf]

A novel cellulose-supported polymer electrolyte with high ionic conductivity for lithium metal batteries

Xuefei Cao[1], Mingyang Xin[*, 1], Jiaxin Yin[2]

[1] *Criminal Investigation and Counter-Terrorism College, Criminal Investigation Police University of China,110854, China*

[2]*School of Chemistry, Northeast Normal University, Changchun, 130024, China*

[*]*Correspondingce: xinmy608@nenu.edu.cn*

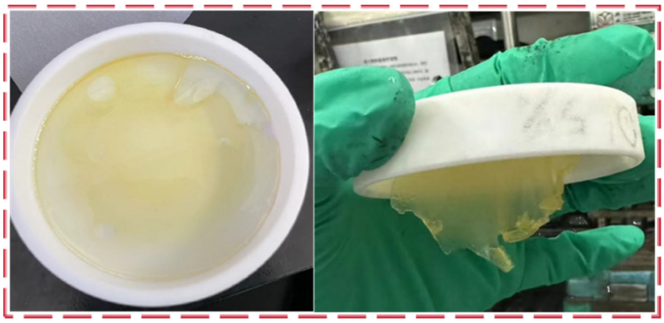

Figure S1. Polymerization of PAAA without a cellulose membrane.

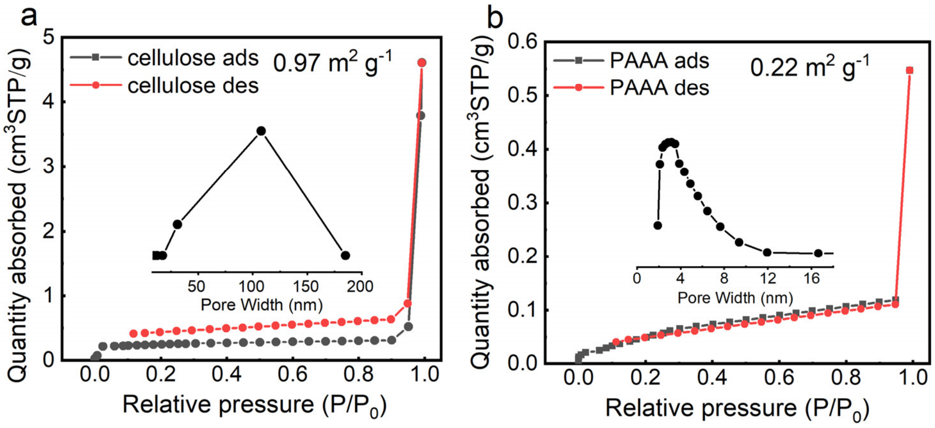

Figure S2. Nitrogen adsorption isotherm and desorption isotherm for (a) cellulose films, (b) PAAA.

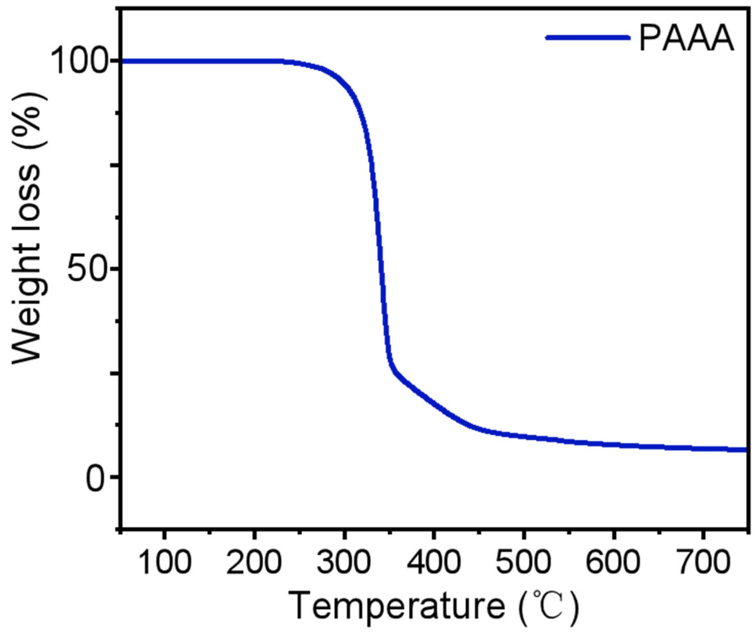

Figure S3. Thermogravimetry curves of PAAA.

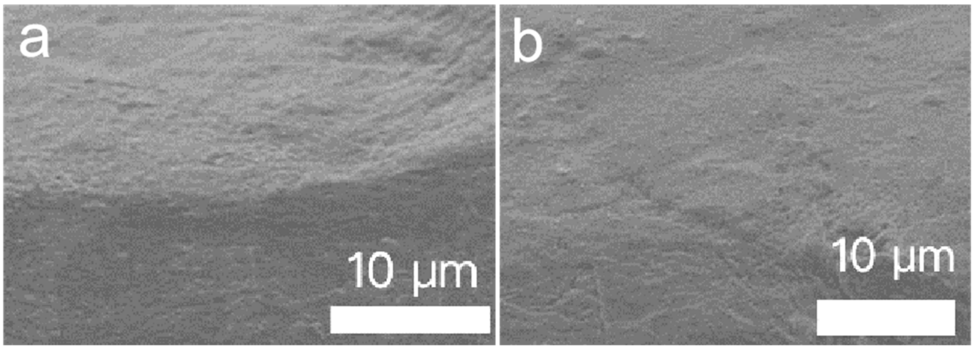

Figure S4. The SEM images from Li-Li cells after cycling.

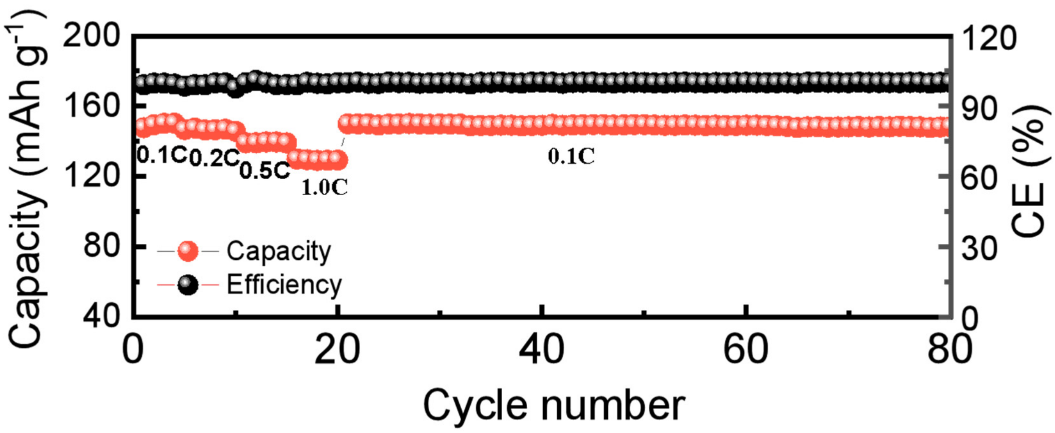

Figure S5. Capacity-efficiency plots of Li‖PAAA‖LFP at various rates.

Equation S1.

$$\delta = \frac{L}{R \times S} \qquad (S1)$$

where δ, L, R and S are the ionic conductivity, the thickness between the steel sheets, the electrochemical impedance and the contact area between the electrolyte and the blocking electrode, respectively.

Equation S2.

$$\sigma = AT^{-\frac{1}{2}} e^{-\frac{Ea}{R(T-T_0)}} \qquad (S2)$$

where A, E_a, R and T_0 are the prefactor, apparent activation energy, ideal gas coefficient and ideal glass transition temperature, respectively.
